# Effectiveness of an educational intervention in enhancing end-of-life care understanding and decision-making in African Americans

**DOI:** 10.1017/S147895152500046X

**Published:** 2025-07-03

**Authors:** Delicia Pruitt, Megan Reilly, Stephen Zyzanski, Neli Ragina

**Affiliations:** 1 CMU College of Medicine, Mount Pleasant, MI, USA; 2 Department of Family Medicine, CMU Educational Partners, Saginaw, MI, USA

**Keywords:** African American, End-of-life (EOL) choices, Education, Palliative care, Hospice care

## Abstract

**Objectives:**

To develop an effective, targeted educational intervention that can serve as a teaching tool to educate African American (AA) populations, especially the elderly, on end-of-life (EOL) options prior to critical care.

**Methods:**

A survey was used to assess the level of preparation and determine deficits in knowledge regarding EOL choices in the AA community of Saginaw, Michigan, before and after educational intervention. We used a paired-sample *t*-test to assess changes in understanding about EOL planning options, McNemar’s to test changes in intention to use hospice and palliative care, and Spearman correlations to identify demographics influencing change of outcomes. Outcome scores associated with multiple demographic variables were regressed on these demographics.

**Results:**

Our data indicated that the intervention was an effective teaching tool in educating the AA population on EOL choices. Significant changes were observed in understanding of EOL options, concerns about palliative and hospice care, and intention to use palliative and hospice care. Age and education were also associated with selected outcome changes.

**Significance of results:**

AA patients are more likely than other ethnic groups to choose life-sustaining measures at the end of their lives, leading to patients not receiving care to help them die peacefully. This decision is partly based on lack of knowledge of the available EOL care options. This study provides evidence needed for physicians to increase their educational efforts with the AA population regarding EOL options. An educational tool like the one developed in this study may be helpful and lessen the time of education so that physicians can answer questions at the end of the session and empower individuals and communities to take an active role in creating a culture of wellness at the EOL and decreasing morbidity.

## Introduction

Advanced care planning (ACP) and end-of-life (EOL) care can help provide patients with dignified, comfortable, and patient-centered care that aligns with their desires (Catlett and Campbell 2021). ACP encompasses a wide range of planning activities including, but not limited to, advanced directives (AD), identifying power of attorney, and decisions on use of palliative or hospice care. The process aims to reduce pain and suffering at the EOL while honoring a patient’s wishes if they cannot make decisions on their own. This is accomplished through weighing future care options with the patient, family, clinicians, and others close to the patient then communicating and documenting preferences for care before a time of crisis (Belisomo 2018; Catlett and Campbell 2021; Sloan et al. 2021). ACP leading to quality EOL care has been shown to improve satisfaction with care as well as ease the decision-making burden on loved ones (Ejem et al. 2019; Sanders et al. 2019).

With the population of older U.S. adults growing rapidly, especially in the African American (AA) community with an expected increase of 115% by 2030, planning for EOL care is imperative to patients’ health and well-being (Ejem et al. 2019). Only one-third of U.S. adults have participated in ACP, and in the AA population, rates are significantly lower (Belisomo 2018; Catlett and Campbell 2021; Collins 2019; Ejem et al. 2019). Studies have shown that the AA community tends to choose life-extending options when faced with a terminal condition as opposed to care focused on comfort and quality of life (Catlett and Campbell 2021; Ornstein et al. 2020; Portanova et al. 2017). They are more likely to not have ACP documents in place with less than 20% having an AD, compared to 51% in white population (Catlett and Campbell 2021; Portanova et al. 2017). They have also been documented to have higher rates of intensive care at the EOL with AA patients receiving less hospice care and more ED visits and hospitalizations than white patients (Ornstein et al. 2020).

Many barriers to EOL care in the AA population have been described in the literature including reluctance to discuss the topic of dying both by physician and patient, lack of knowledge on ACP and EOL care, mistrust in healthcare systems, and religious beliefs about death and dying (Belisomo 2018; Collins 2019; National Institute on Aging (NIA) 2017; Sanders et al. 2019; Sloan et al. 2021). Overcoming many of these barriers requires major systematic change and increased ACP and EOL care understanding has been shown to be a promising step toward improved EOL care (Belisomo 2018; Catlett and Campbell 2021; Sanders et al. 2019). By having knowledge of the ACP process and all options for EOL care, patients are able to make informed decisions about their health care that is in accordance with their belief systems.

A lack of knowledge of ACP and EOL care has been well documented in the AA population (Belisomo 2018; Catlett and Campbell 2021). Studies have found that education about options and engaging in respectful communication may facilitate ACP and lead to improved EOL care (Belisomo 2018; Sanders et al. 2019). In 2020, a pilot study was conducted that provided an educational intervention with information on ACP and EOL including AD, wills, power of attorney, and palliative and hospice care. It showed the intervention was successful in improving knowledge and understanding of ACP as well as intent to implement documents for EOL care and use palliative and hospice care. The intervention also alleviated concerns surrounding these topics (Pruitt et al. 2021). This study’s aim is to use educational interventions in the form of information on ACP and EOL care topics and open discussion with a larger sample size to provide a more generalizable and stronger conclusion on the efficacy of educational intervention on understanding of and intent to use EOL care planning.

## Methods

### Participants

This study consisted of 138 participants from Saginaw, Michigan. Participants were recruited from local churches. The churches were called to determine their interest in participating in the study. Institutional Review Board-approved flyers were distributed and posted at each interested church with the dates and times of the study. All potential participants attended church in-person or online on the day of the study. For in-person participants, the surveys and presentation were administered in a separate location than the church service, like the gym or basement, to ensure voluntary participation. During the pandemic, church participants attended online prior to the church service. Only those volunteers who were interested in the study attended the online presentation. All participants received a gift card for their participation.

### Survey design

Pre- and post-intervention surveys were designed to assess participants’ knowledge and perception regarding the following: (a) knowledge regarding documents that can be put in place to assist during EOL decision making such as a will, AD, life insurance; (b) awareness of power of attorney and if they are already in process of designating one; (c) confidence in their understanding regarding wills, AD, power of attorney, life insurance, hospice care, and palliative Care; (d) consideration of palliative care use if one is given a terminal diagnosis (told that he/she is going to die) and if no or not decided what the underlying reason for this is (it costs too much, faith in God, there will be less money for others in my family, mistrust such as hospice care will give permission to the doctors to hasten my death, or others).

The pre-intervention survey also included demographic questions (see Table 1). Additional questions solicited information regarding the current status of the participants EOL preparation such as: “I am in the process of having a will written up,” “I have told a family member and/or a doctor about my wishes regarding a will, but they are not documented.”

**Table 1.** Demographic information of participants overall, with will in place, and with advanced directive in place

	Overall (*N* = 136)	Will in place (*N* = 40)	Advanced directive in place (*N* = 24)
Age			
18−30	12 (8.8%)	2 (8.8%)	0
30−59	66 (48.5%)	17 (42.5%)	10 (41.7%)
60+	58 (42.8%)	21 (52.5%)	14 (58.3%)
Sex			
Female	40 (29.4%)	14 (35%)	7 (29.2%)
Male	95 (69.9%)	26 (65%)	17 (70.8%)
Chronic Conditions			
Diabetes	25 (18.4%)	8 (20%)	7 (29.2%)
Hypertension	59 (43.4%)	17 (42.5%)	11 (45.8%)
Coronary artery disease	3 (2.2%)	1 (2.5%)	0
COPD	3 (2.2%)	1 (2.5%)	1 (4.2%)
Congestive heart disease	1 (0.7%)	0	0
Dementia	2 (1.5%)	1 (2.5%)	1 (4.2%)
Other	15 (11%)	4 (10%)	1 (4.2%)
Education			
High school	58 (42.6%)	15 (37.5%)	8 (33.3%)
Associates	24 (17.6%)	12 (30%)	11 (45.8%)
Bachelor’s	26 (19.1%)	8 (20%)	3 (12.5%)
Master’s	19 (14%)	5 (12.5%)	2 (8.3%)
PhD/MD	2 (1.5%)	0	0
Trade school	7 (5.1%)		
Has in place…			
Will	40 (29.4%)		16 (66.7%)
Advanced directive	24 (17.6%)	16 (40%)	
Power of attorney	41 (30.1%)	27 (67.5%)	18 (75%)
Life insurance	107 (78.7%)	36 (90%)	22 (91.7%)

The post-intervention survey was the same as the pre-intervention survey with the demographic questions removed. Questions on both survey’s measuring changes in understanding utilized a 6-point Likert scale varied from 1 = Strongly Disagree to 6 = Strongly Agree. Change in attitude toward using palliative or hospice care utilized1 = “No” or 2 = “Yes” answer options. To link the pre- and post-intervention surveys, both surveys asked participants to provide the first 2 letters of the name of the street on which they grew up, the first 2 numbers of the earliest phone number they can remember, and the first 2 letters of mother’s maiden name. The Central Michigan University Institutional Review Board Committee approved the study.

### Teaching tool design (educational intervention)

The teaching tool was designed with the guidance of a counselor who has had counseling experience with AA patients and understands the belief system of the AA population. After 2 sessions, the counselor and Primary Investigator developed the steps of preparing for the EOL. Information on hospice and palliative care was obtained from the NIH website. The average expenses for funerals in Michigan, information about wills and assets were obtained by a Google search. A video lasting 10 min was designed to present this information, or the participants were given a live talk virtually from Dr. Delicia Pruitt with the same information. Pre-surveys were collected before the educational intervention and post survey were collect post the educational intervention.

### Statistics analysis

The statistical analyses were done in steps. In step 1, descriptive statistics were computed to provide a profile of the study sample’s categorical demographics and current health status. Next, analyses of change, pre/post, were computed for 3 sets of outcomes. The outcomes included the level of understanding of five EOL options, the number of concerns about palliative and hospice care, and the subject’s intention to use palliative and hospice care. Each pre/post mean change was tested by paired *t*-test and McNemar’s statistic was used to test for changes in intention to use hospice and palliative care. Next, Spearman correlations were used to identify whether a subject’s age, gender, or educational level were related to any of the EOL change outcomes. Lastly, for any outcome change associated with more than 1 demographic variable, outcome scores were regressed on the multiple demographics to determine if each demographic variable had an independent contribution to predicting positive change in outcome.

## Results

The demographic profile of the participants is described in Table 1. All our participants were AA. Almost half of them experienced a chronic condition with 18% being diabetic and 43% hypertensive. Forty-eight percent of our subjects were between 30 and 59 years of age and 42.8% were 60 years of age or over. Almost half of the subjects indicated that high school was the highest educational degree achieved. Forty (30%) of our participants had a will, 24 (17%) had advance directives, 41 (30%) had power of attorney, and almost all the participants (79%) had life insurance. To better understand the sample, we examined the demographic of the participants who had a will in place (Table 1). Half of the participants who had a will in place were over 60 years of age, over 60% had diabetes and/or hypertension, just under 40% only had a high school degree, and 90% of them had life insurance in place as well. Table 1 also outlines the demographic information for the 24 participants who indicated they have AD in place. This group indicated higher educational attainment than those with wills. In addition to specifying AD, they also were more likely to have a will, power of attorney, and life insurance in place.

Paired sample *t*-tests revealed the educational intervention was effective in improving knowledge (Table 2). Participants reported their understanding of wills significantly improved from pre-intervention to the post intervention assessment, *p* < .001. Similarly, understanding of advance directives significantly improved from the pre-intervention to the post intervention, *p* < .001. Understanding of power of attorney also significantly improved from the pre-intervention to the post intervention, *p* < .001. Additionally, understanding of palliative care significantly improved from the pre-intervention to the post intervention, *p* < .001. Likewise, understanding of hospice care significantly improved from the pre-intervention to the post-intervention, *p* < .001. Understanding about life insurance also significantly improved from the pre-intervention to the post intervention even though the majority (79%) already had life insurance (*p* < 0.05).

**Table 2.** Pre- and post-intervention mean item score for understanding of will, advanced directive, power of attorney, life insurance, and palliative and hospice care

	Pre-intervention mean	Post intervention mean	Paired *t*-value	SD of diff.
Will (*N* = 110)	4.75	5.23	4.82***	1.03
Advance directive (*N* = 104)	3.61	5.03	5.91***	1.59
Power of attorney (*N* = 109)	4.64	5.21	4.91***	0.92
Life insurance (*N* = 112)	5.05	5.28	2.33*	0.77
Palliative care (*N* = 93)	2.84	4.7	8.45***	1.89
Hospice care (*N* = 108)	4.41	5.1	4.05***	1.38

* 0.05;

*** <0.001.

The educational intervention also helped to ease concerns about palliative care and hospice care (Table 3). Concerns about palliative care decreased from the pre-intervention to the post- intervention (*p* < .001). Likewise, concerns about hospice care decreased from the pre-intervention to the post intervention (*p* < .001).

**Table 3.** Pre- and post-intervention mean survey score for concerns about palliative and hospice care

	Pre-intervention mean	Post-intervention mean	Paired *t*-value	SD of diff.
Concerns about palliative care (*N* = 121)	0.74	0.36	5.35***	0.76
Concerns about hospice care (*N* = 122)	0.64	0.34	4.29***	0.78

*** < 0.001.

In addition to improved knowledge and ease of concerns, the intervention resulted in intended behavioral change (Table 4). Considering use of palliative care significantly increased from the pre-intervention to the post intervention assessment (*p* < 0.0010. Similarly, considering use of hospice care increased from the pre-intervention to the post-intervention (*p* < 0.001).

**Table 4.** Pre- and post-intervention intention to use hospice and palliative care

	Pre-intervention	Post-intervention	Significance
Intention to use hospice care (*N* = 123)	60 (49%)	95 (77%)	<0.001*
Intention to use palliative care (*N* = 117)	29 (25%)	80 (68%)	<0.001*

* McNemar’s test.

Associations between the three demographic variables of age, gender, and education and the study outcomes were examined using Spearman correlations. Only a few statistically significant associations were observed. Older subjects were significantly more likely to have an AD (*r* = 0.17, *p* = 044) and a power of attorney (*r* = 0.28, *p* = 0.001). Female subjects were more likely to have life insurance (*r* = 0.17, *p* = 043). Younger subjects significantly improved their understanding of palliative care (*r* = − 0.24, *p* = 0.019), increased their intention to use hospice care (*r* = −0.21, *p* = 0.022), and decreased the number of their concerns about palliative care (*r* = –0.31, *p* = 0.001). Subjects with more education showed increased understanding of palliative care post intervention (*r* = 0.27, *p* = 0.007). Since both age and education were associated with increased understanding of palliative care, a regression analysis was performed to determine if these were independent effects (Table 5). The results show that both younger age and greater levels of education were independently associated with increased understanding of palliative care. Younger, better educated subjects derived the most benefit from the intervention in terms of their understanding of palliative care.

**Table 5.** Regression of improved post-intervention understanding of palliative care on age and education

Variables	*B*	S.E.B	Beta	*t*-Test	*p*-Value
Age	−0.74	0.3	−0.24	2.45	0.016
Education	0.37	0.17	0.22	2.17	0.033

## Discussion

Our study with an increased sample size again demonstrated that educational intervention is successful in educating the AA population on ACP and EOL care. From pre to post survey, there were statistically significant increases in understanding of ACP including wills, AD, power of attorney, life insurance, and palliative and hospice care. The education also resulted in the intended outcome of easing concerns surrounding palliative and hospice care with participants expressing increased intention to use these services.

When considering demographics, data showed that participants who had a will were significantly more likely to also have other ACP documents such as AD, power of attorney, and life insurance. Neither gender nor having a chronic disease (diabetes, hypertension, etc.) were associated with having a prior will or AD. We may conclude that once ACP is initiated, people tend to continue the process of documenting their wishes fully by completing multiple ACP activities. However, having a chronic disease does not increase likelihood of having documents in place. This could be due to lack of conversation about ACP due to apprehension about discussing death and dying both by the physician and patient. The disease categories offered in our questionnaire covered many nonterminal diseases and the patients may not have been prompted to discuss EOL care with these diagnoses. Further studies should be directed into looking at nonterminal and terminal diagnoses and EOL care use.

Older participants were significantly more likely to have ACP documents such as AD and POA in place, while younger participants were more likely to significantly improve in knowledge of palliative care and intention to use hospice care. These age factors are likely due to timing of conversations about EOL planning with older adults having already initiated discussion of EOL care and younger adults having less education about ACP. These findings suggest that early EOL care education could be beneficial for future ACP and intention to use EOL care, and are in concordance with the pilot study (Pruitt et al. 2021) as well as other recent studies that showed education and open, respectful conversations about ACP and EOL lead to increased knowledge and intent to use these services (Belisomo 2018; Catlett and Campbell 2021; Sanders et al. 2019). The results of this study show that implementing educational programs and interventions can be an effective way to increase understanding of and intention to use EOL planning in the AA community.

Although we did not assess actual implementation of ACP documents or use of palliative and hospice care, increasing understanding and awareness of options will allow people to make informed decisions regarding their care in accordance with their personal beliefs and wishes. Other studies have explored the use of faith-based education about EOL and found that these approaches also lead to increases in knowledge and openness to EOL planning and care but did not increase AD completion in the population (Catlett and Campbell 2021). It is possible that the driving force behind lack of use of EOL care are other factors such as cultural and religious beliefs, mistrust in health care, etc. However, the significant increase in intent to use ACP and EOL care in our findings suggests that this intervention may increase EOL care use. Further studies should be conducted to assess participants’ completion of EOL documents and use of palliative and hospice care, as well as explore more reasons for lack of utilization of ACP and EOL care options.

### Study limitations

The study was limited in that we did not follow the participants long term to determine if the educational intervention resulted in participants actually making end-of -life choices, such as living wills, AD, power of attorney, establishing life insurance, or planned for hospice or palliative care. Future studies can be focused on discerning patients’ execution of intentions to use ACP and EOL care. Because our study was primarily focused in Saginaw county, the study results may not be translatable to AAs in other counties in Michigan. Future work will focus on further expanding the study and educational intervention beyond the boundaries of Saginaw County. Finally, in this study, 35% of the participants were college educated with a bachelor’s or higher degree. In Saginaw in 2022, 13% of AAs had a bachelor or higher degree. The participants, therefore, may not be a cross-sectional representation of the Saginaw community. Future studies that incorporate different churches and will be done at public venues to get a more representative cross-sectional sample of Saginaw, Michigan’s AA population.

## References

[ref1] Belisomo R (2018) Reversing racial inequities at the end of life: A call for health systems to create culturally competent advance care planning programs within African American communities. *Journal of Racial and Ethnic Health Disparities* 5, 213–220. doi:10.1007/s40615-017-0360-2 28409478

[ref2] Catlett L and Campbell C (2021) advance care planning and end of life care literacy initiative in African American faith communities: A systematic integrative review. *American Journal of Hospice & Palliative Medicine* 38(6), 719–730. doi:10.1177/1049909120979164 33297716

[ref3] Collins J (2019) *Cultural Aspects of End of Life Advance Care Planning for African Americans: An Ethnonursing Study*. Duquesne University. Doctoral dissertation. 10.1177/1043659620960788 32988287

[ref4] Ejem DB, Barrett N, Rhodes RL, et al. (2019) Reducing disparities in the quality of palliative care older African Americans through improved advance care planning: Study design and protocol. *Journal of Palliative Medicine* 22(1), 90–100. doi:10.1089/jpm.2019.0146 31486728

[ref5] National Institute on Aging (NIA) (2017) What Are Palliative Care and Hospice Care? www.nia.nih.gov.

[ref6] Ornstein KA, Roth DL and Huang J (2020) Evaluation of racial disparities in hospice use and end-of-life treatment intensity in the regards cohort. *JAMA Network Open* 3(8), e2014639. doi:10.1001/jamanetworkopen.2020.14639 PMC7445597 32833020

[ref7] Portanova J, Ailshire J, Perez C, et al. (2017) ethnic differences in advance directive completion and care preferences: What has changed in a decade?. *Journal of the American Geriatrics Society* 65(6), 1352–1357. doi:10.1111/jgs.14800 28276051 PMC5893138

[ref8] Pruitt D, Weber K and Ragina N (2021) Teaching end-of-life preparation to African Americans. *Palliative & Supportive Care* 19(3), 335–340. doi:10.1017/S1478951520001078 33155536

[ref9] Sanders JJ, Johnson KS, Cannady K, et al. (2019) From barriers to assets: Rethinking factors impacting advance care planning for African Americans. *Palliative & Supportive Care* 17, 306–313. doi:10.1017/S147895151800038X 29869594

[ref10] Sloan DH, Gray TF, Harris D, et al. (2021) Church leaders and parishioners speak out about the role of the church in advance care planning and end-of-life care. *Palliative & Supportive Care* 19, 322–328. doi:10.1017/S1478951520000966 33118897

